# Controlled Antibiotic Release From Emulsion-Loaded Alginate and Fibrin Hydrogels Using Ultrasound

**DOI:** 10.1002/jbma.70046

**Published:** 2026-02

**Authors:** Ziba Ghareh Nazi Fam, Asia Winslow, Mario L. Fabiilli, Sam Varghese, Brian E. Oeffinger, Flemming Forsberg, Noreen J. Hickok, Lauren J. Delaney

**Affiliations:** 1 Department of Radiology, Thomas Jefferson University, Philadelphia, Pennsylvania, USA; 2 Department of Orthopedic Surgery, Thomas Jefferson University, Philadelphia, Pennsylvania, USA; 3 Department of Radiology, University of Michigan, Ann Arbor, Michigan, USA

**Keywords:** alginate, controlled release, fibrin, phase-shift emulsions, ultrasound

## Abstract

Postoperative infections remain a major complication after spinal fusion surgery, often caused by biofilm-forming bacteria that resist short-term antibiotic prophylaxis. Vancomycin (VAN) is sometimes delivered locally during surgery; however, levels diminish rapidly, leaving patients vulnerable to late-onset infections. We developed a composite hydrogel integrating perfluorohexane-based emulsions within alginate or fibrin matrices to enable both sustained and ultrasound-triggered VAN release. Water-in-oil-in-water emulsions were prepared using fluorosurfactant-stabilized perfluorohexane as the volatile oil phase, then embedded in hydrogels and exposed to ultrasound (2.5 MHz, 5.5 MPa peak negative pressure) to initiate acoustic droplet vaporization for triggered release. Drug release was quantified spectrophotometrically and with fluorescent-labeled VAN, while antibacterial efficacy was tested against *Staphylococcus aureus*. Hydrogels directly loaded with free VAN exhibited burst release (~55%–67% within 24 h) followed by limited sustained release, which is suboptimal for prolonged coverage. In contrast, emulsion-loaded hydrogels reduced premature leakage, retaining > 70% VAN on Day 1 and providing gradual baseline release. Ultrasound application enhanced VAN release up to 8.75-fold in alginate and 27.5-fold in fibrin hydrogels after 7 days (*p* < 0.0001), supporting both continuous and on-demand delivery. Only alginate-emulsion hydrogels showed measurable antibacterial activity, as drug-matrix interactions in fibrin prevented release; ultrasound-treated samples displayed significantly greater efficacy over 7 days (*p* < 0.05 vs. no ultrasound). This dual-mode delivery platform enables spatial and temporal control of VAN release, combining early prophylaxis with ultrasound-triggered reinforcement, and holds promise for improving infection prevention in orthopedic surgeries by aligning drug delivery with clinical timelines.

## 1 ∣ Introduction

Bacterial infection following spinal fusion surgery remains a significant clinical concern, with 1%–10% of patients developing infections despite the use of aggressive perioperative antibiotic treatment [1-3]. Placing approximately 1–2 g of powdered vancomycin (VAN) along with aseptic drains at the surgical site has been used in many cases to reduce the risk of infection during wound closure [4, 5]. While this prophylactic measure provides short-term protection (typically 24–48 h), it may be insufficient in a small proportion of cases (up to 20%), where certain types of bacteria persist within the surgical cavity [6, 7]. These bacteria, often capable of forming biofilms, can survive initial antibiotic exposure and colonize implant surfaces. Once biofilms are established, they significantly increase the risk of infection and complicate treatment, often requiring prolonged systemic antibiotic therapy and, in severe cases, surgical removal of the infected hardware [8]. This significantly extends hospitalization and patient recovery time. These limitations highlight the need for more effective methods to prevent infections, particularly those that can combat pathogens before they adhere to spinal hardware.

Current treatments, primarily involving local VAN powder and short-term systemic antibiotics, often fail to provide lasting protection once the initial prophylactic antibiotics dissipate [9, 10]. To address this, there is an urgent need for novel drug delivery systems that can sustain therapeutic levels of antibiotics and/or antimicrobials at the surgical site after an extended period (7+ days). This approach would, crucially, provide extended coverage against surviving bacteria after drain removal and depletion of the initial peri-operative antibiotics. Hydrogels, three-dimensional polymer networks capable of holding large amounts of water, have been widely explored as local drug delivery depots due to their ability to encapsulate, protect, and release therapeutics in a controlled manner. In this context, an implantable hydrogel that not only augments the current antimicrobial prophylaxis but also extends its duration of action through controlled and bolus antibiotic release is needed. This hydrogel should release VAN in a sustained manner during the period when the surgical drains are in place, providing ongoing prophylaxis, and allow external ultrasound stimulation to trigger the release of complementary antibiotics upon drain removal to attack any remaining pathogens [11-15].

Several previous studies have explored stimuli-responsive hydrogels for controlled antibiotic delivery [16-18]. For example, near-infrared light has been employed to trigger antibiotic release from injectable hydrogels composed of polydopamine nanoparticles and glycol chitosan [19]. Other approaches have utilized pH- and temperature-sensitive hydrogels containing silver nanoparticles for rapid bacterial elimination [20], or pH-responsive smart hydrogels incorporating aminoglycoside antibiotics, pectinase, and oxidized dextran to treat biofilm-associated infections [21]. While Delaney et al. [15, 22] have successfully demonstrated ultrasound-responsive antibiotic release from implantable reservoirs using polylactic acid-coated PEEK systems, such devices remain rigid and surgically anchored, limiting their adaptability and use across tissue types. Hydrogels, by contrast, offer flexible, injectable, and bioresorbable platforms that may be more suitable for covering irregular anatomical spaces and/or soft-tissue interfaces.

Ultrasound-triggered antibiotic release has also been demonstrated in chitosan/ethylene glycol diglycidyl ether hydrogels loaded with amoxicillin. In this study, ultrasound enhanced amoxicillin release mainly by softening the hydrogel matrix and promoting non-Fickian drug diffusion through mechanical effects at 43 kHz [23]. Additionally, hybrid hydrogels composed of sodium alginate, sucrose acetate isobutyrate, and electrospray microspheres have shown promise for ultrasound-responsive, on-demand drug release. Ultrasound can trigger drug release by breaking ionic crosslinks, temporarily loosening the hydrogel network and allowing faster diffusion, with release slowing once ultrasound stops [24]. A particularly promising ultrasound-responsive strategy involves the incorporation of phase-shift emulsions into the hydrogel matrix. Upon insonation above a certain pressure threshold, these liquid droplets rapidly vaporize into gas microbubbles, a process termed acoustic droplet vaporization (ADV), thereby accelerating drug release. This mechanism enables spatiotemporally controlled, on-demand delivery while retaining minimal release in the absence of ultrasound, offering a tunable platform for antibiotic release at infection sites [25-27].

Both alginate and fibrin have independently been explored as hydrogel scaffolds due to their biocompatibility, tunable gelation properties, and potential for drug encapsulation [28, 29]. Furthermore, multiple studies have investigated their use for localized antibiotic delivery in surgical site infections, including those of the spine [30-32]. Additionally, these hydrogels offer tunable crosslinking and degradation properties, allowing their release profiles to be tailored for different clinical scenarios, ranging from fast, short-duration delivery to prolonged, controlled release over time. Alginate, a polysaccharide derived from brown algae, forms ionically crosslinked networks that enable sustained release of hydrophilic drugs. However, it suffers from poor mechanical integrity and limited bioactivity. Importantly, recent studies have demonstrated that alginate hydrogels can also serve as acoustically responsive matrices enabling ultrasound-triggered drug release [24, 33, 34]. Fibrin, in contrast, is a natural protein-based polymer that supports cell infiltration and degradation in vivo, making it attractive for regenerative applications and wound healing. Notably, fibrin has also been shown to form acoustically responsive scaffolds (ARS) when combined with phase-shift emulsions, enabling ultrasound-mediated drug delivery [25]. However, the release kinetics, structural stability, and mechanical behavior of these hydrogels can vary widely depending on their composition and application.

While previous systems have demonstrated the potential of stimuli-responsive hydrogels for antibiotic delivery, most are limited to either passive sustained release or externally triggered release, without offering precise control over both in a clinically adaptable format. Furthermore, rigid or surgically anchored devices, such as polylactic acid-coated PEEK reservoirs, lack the flexibility needed for soft tissue applications. In contrast, injectable hydrogels offer a more versatile platform, capable of conforming to irregular anatomical spaces and degrading safely in vivo. Hence, we selected alginate or fibrin-based hydrogels and compared their suitability for our dual-mode antibiotic delivery system. While other ultrasound-triggered drug delivery systems often rely on mechanisms such as hydrogel matrix softening to enhance drug diffusion or disruption of ionic crosslinks to promote release, our approach incorporates perfluorohexane-based emulsion into both alginate and fibrin matrices to enable sustained passive VAN release and externally triggered, on-demand release via ADV. In this method, ADV disrupts the morphology of the phase-change droplet vaporization, thereby facilitating rapid drug release. By systematically evaluating and comparing these formulations, this study aims to determine which hydrogel provides the best spatiotemporal control over antibiotic release and antimicrobial efficacy, advancing the development of adaptable drug delivery platforms for infection prevention in spinal fusion and potentially other postsurgical applications.

## 2 ∣ Materials and Methods

### 2.1 ∣ Emulsion Preparation

To synthesize the fluorosurfactant block copolymer (FSH-PEG-FSH), a 2:1 M ratio of Krytox 157 FSH (Miller-Stephenson, Danbury, CT, USA), which contains a terminal carboxylic acid group, and poly(ethylene glycol) bis(amine) (PEG, MW: 1000, Thermo Fisher Scientific, Waltham, MA, USA) was used [35]. Primary water-in-oil (W_1_/O) emulsions were formed using a Q55 sonicator with a CL-188 immersion probe (QSonica, Newtown, CT, USA). The inner aqueous phase (W_1_) was prepared by dissolving VAN (Pfizer, New York, NY, USA) at 50 mg/mL in deionized water. The VAN solution was emulsified into perfluorohexane (Strem Chemicals, Newburyport, MA, USA) containing 2% (w/w) FSH-PEG-FSH, resulting in a 1:2 (v/v) ratio of aqueous to oil phases. FITC-labeled vancomycin (FITC-VAN, Nanocs, Boston, NY, USA), dissolved at 2 mg/mL in the W_1_ phase, was used as a fluorescent tracer in some formulations. Coarse double emulsions (W_1_/O/W_2_) were generated by adding the primary emulsion dropwise to a 50 mg/mL solution of Pluronic F68 in phosphate-buffered saline that was stirred at 700 rpm for 2 min. The double emulsion was placed on ice, and the particle size was reduced using an immersion blender (T10, IKA Works, Wilmington, NC, USA). The average diameter of the VAN and FITC-VAN emulsions was 1.70 ± 0.18 μm and 1.82 ± 0.22 μm, respectively.

In this system, VAN is encapsulated within the inner aqueous phase (W_1_) of a water-in-oil-in-water (W_1_/O/W_2_) double emulsion. The intermediate oil layer, containing volatile perfluorohexane, undergoes ADV upon exposure to ultrasound exceeding a certain acoustic pressure threshold. This process, initiated during the negative pressure phase of the ultrasound wave, causes a liquid-to-gas phase transition that disrupts the emulsion structure and triggers drug release [25].

### 2.2 ∣ Hydrogel Preparation

Low-viscosity, 10 mg/mL alginate hydrogels were prepared by first dissolving sodium alginate (Sigma-Aldrich, St. Louis, MO, USA) in deionized water. Prior to use, the sodium alginate solution was degassed for 10 min to minimize the amount of dissolved gas that could interfere with insonation. Ionic crosslinking was achieved by adding calcium carbonate (CaCO_3_) and glucono-δ-lactone (GDL) at final concentrations of 20 and 40 mM, respectively. These crosslinker concentrations were selected to allow incorporation of the drug without causing rapid gelation. After adding VAN or VAN-loaded emulsions to the alginate solution, gelation occurred over approximately 1 min. Two VAN loading mechanisms were evaluated in this study: direct loading into the hydrogel matrix and encapsulation within emulsion droplets. For direct loading, the VAN solution was incorporated into the alginate solution prior to polymerization to achieve a VAN loading of 0.25 mg per hydrogel. For emulsion-based loading, VAN or FITC-VAN emulsions were incorporated at 1.5% (v/v) in hydrogels of 1 mL or 0.5 mL volume, respectively.

Fibrin hydrogels were prepared by dissolving bovine fibrinogen (Sigma-Aldrich, St. Louis, MO, USA) in FluoroBrite Dulbecco′s modified Eagle′s medium (DMEM) and then degassing the solution. VAN solution and emulsions were incorporated into 5 mg/mL fibrin hydrogels similarly to the alginate hydrogels. Fibrin hydrogels contained 0.025 U/mL bovine aprotinin (Sigma-Aldrich, St. Louis, MO, USA) and were polymerized with 2 U/mL thrombin (King Pharmaceuticals, Bristol, TN, USA). The dimensions of all hydrogels, which were polymerized in 24-well BioFlex plates (FlexCell International, Burlington, NC, USA), were 15.6 mm (diameter) × 5.2 mm (height). Following polymerization, hydrogels were covered with 1 mL of Dulbecco′s phosphate-buffered saline (DPBS) with calcium and magnesium.

### 2.3 ∣ Hydrogel Insonation for Ultrasound-Triggered Release

Ultrasound-triggered release was performed in a water tank containing degassed, deionized water at 37°C. For release experiments, the BioFlex plates containing hydrogels were held in place on the surface of the water, with only the bottom surface in contact with the water to both preserve sample integrity and provide an acoustic window. A single-element, focused ultrasound transducer (2.5 MHz, H-108, f-number: 0.83, radius of curvature: 50 mm, Sonic Concepts, Bothell, WA, USA) was used to induce ADV. The transducer was calibrated in a free-field setup using a custom fiber optic hydrophone (105 μm, AIMS III, Onda, Sunnyvale, CA, USA). Pulsed signals were generated by a dual-channel function generator (33500B, Agilent Technologies, Santa Clara, CA, USA), amplified with a gated RF amplifier (GA-2500A, Ritec Inc., Warwick, RI, USA), and transmitted through a matching circuit to reduce impedance mismatch. Signal output was verified using a digital oscilloscope (HDO4034, Teledyne LeCroy, Chestnut Ridge, NY, USA). Standard acoustic parameters included a 5.5 MPa peak negative pressure, 12 μs pulse duration, and 100 Hz repetition frequency (duty cycle: 0.12%; mechanical index: 3.48) based on our prior study [25]. The transducer was mounted below the plate and aligned using a motorized 3-axis stage controlled via MATLAB (MathWorks, Natick, MA, USA). The axial focus was adjusted using a pulse-echo alignment method with a pulser/receiver unit (5077 PR, Olympus, Center Valley, PA, USA), based on reflections from the well bottom. Insonation was applied at focal depths of 1, 3, or 5 mm above the well base, with the transducer rastered laterally at 5 mm/s until the entire hydrogel was insonified.

### 2.4 ∣ Release Monitoring

On Day 0, 15 min after hydrogels were immersed in medium, the medium was completely collected from each well and replaced with fresh medium. This sampling procedure was repeated on Days 1, 3, and 7 to evaluate cumulative VAN release. A subset of alginate and fibrin hydrogels with emulsion was subjected to ultrasound treatment immediately following the Day 0 sample collection to trigger release from the emulsion-loaded hydrogels. Alginate hydrogels were kept in the BioFlex plate for the duration of the study. Due to the larger volumetric expansion that occurred over the study, fibrin hydrogels were transferred to 12-well plates, with each well filled with 2 mL of DPBS to ensure the gels remained fully immersed. A summary of the experimental groups for alginate and fibrin hydrogels is provided in Table 1. After each sampling and insonation step (when applicable), plates were returned to the incubator and maintained at 37°C. VAN concentration in the collected solutions was measured using a SpectraMax M2e microplate reader (Molecular Devices, San Jose, CA, USA) and a Tecan Infinite spectrophotometer (Tecan, Männedorf, Switzerland). Absorbance was recorded at 280 nm, corresponding to the characteristic wavelength of VAN. Release of FITC-VAN was quantified by measuring fluorescence emission at 525 nm with excitation at 495 nm. A mass balance approach was used to calculate release profiles using calibration curves for VAN and FITC-VAN. All measurements were performed with at least four independent replicates (*n* ≥ 4).

### 2.5 ∣ Antibacterial Activity

The antibacterial efficacy of the hydrogels was evaluated using the Kirby–Bauer disk diffusion assay against methicillin-susceptible *Staphylococcus aureus* (MSSA, ATCC 25923). A total of 13 samples were tested, including hydrogel formulations at four time points (before ultrasound triggering, and 1, 3, and 7 days postinsonation), along with a control solution: 0.25 mg/mL VAN solution. Each sample was tested in triplicate for reproducibility. For each test, 50 μL of the sample was applied to a sterile paper disc, which was then placed on agar plates inoculated with the bacterial strain. Plates were incubated overnight at 37°C to allow for bacterial growth. Zones of inhibition (ZOIs) formed around the discs were measured to determine the antimicrobial effect of each sample. ZOI diameters were compared to a standard calibration curve to estimate the VAN concentration and assess drug release efficacy from the hydrogels over time and under different treatment conditions.

### 2.6 ∣ Statistical Analysis

All experiments were performed with a minimum of three independent replicates, unless otherwise specified. Data are reported as mean ± standard deviation (SD). Statistical significance was determined using two-way ANOVA followed by Tukey′s post hoc test for multiple comparisons. For pairwise comparisons, two-tailed Student′s *t*-tests were employed. A *p* < 0.05 was considered statistically significant. Normality of all data sets was confirmed prior to analysis. All statistical analyses were conducted using GraphPad Prism (Version 10.4.2; GraphPad Software LLC, San Diego, CA, USA).

## 3 ∣ Results

### 3.1 ∣ Spectrophotometric Release Measurement

Incremental and cumulative VAN and FITC-VAN release profiles from the six experimental groups are summarized in Figure 1 and Table S1. Each group consisted of either alginate or fibrin hydrogels, with VAN and FITC-VAN delivered via two loading methods: direct (free VAN/FITC-VAN) or encapsulated within emulsion droplets, with or without ultrasound stimulation.

Both alginate and fibrin hydrogels with free VAN loading (Alginate + VAN and Fibrin + VAN) exhibited a strong initial burst release, with 65% ± 15% and 86% ± 21% of VAN released on Day 1, respectively. These groups then transitioned into a slower release phase, reaching complete (100% ± 6%) release in alginate and an apparent 130% ± 24% release in fibrin by Day 7 (Figure 1c). The latter may be attributed to degradation of the fibrin matrix and optical interference in the spectrophotometric measurements, which may have led to an overestimation of drug concentrations.

In contrast, emulsion-loaded hydrogels showed more sustained release kinetics. Among alginate formulations, ultrasound application on Day 1 enhanced release, with Alginate + VAN emulsion + US releasing 29% ± 7% of VAN on Day 1 compared to 20% ± 5% in the nontreated Alginate + VAN emulsion group, although this difference was not statistically significant (*p* = 0.06). By Day 7, the cumulative release in the ultrasound-treated group (Alginate + VAN emulsion + US) reached 73% ± 10%, nearly double that of the untreated group (40% ± 2%), representing a statistically significant difference (*p* < 0.01; Figure 1a,c).

However, fibrin-based emulsion groups did not show a significant difference in drug release between ultrasound-treated (Fibrin + VAN emulsion + US) and untreated (Fibrin + VAN emulsion) samples at either early (*p* > 0.99) or later time points (*p* = 0.82), with both releasing approximately 30% of VAN on Day 1 and reaching 60%–65% cumulative release by Day 7 (Figure 1b,c).

Both alginate and fibrin hydrogels containing free FITC-VAN exhibited an initial burst release on Day 1 (55% ± 2% and 67% ± 4.3%, respectively), followed by sustained release. By Day 7, cumulative release reached 69% ± 2.7% in Alginate + FITC-VAN and 88% ± 5.5% in Fibrin + FITC-VAN. The release from fibrin was significantly higher than that from alginate (*p* = 0.02; Figure 1f).

In alginate hydrogels, ultrasound treatment significantly enhanced VAN release with Alginate + FITC-VAN emulsion + US exhibiting 22% ± 1% release on Day 1 and 35% ± 2% cumulative release by Day 7, compared to only 2.3% ± 0.3% and 4.2% ± 1.2%, respectively, in the nonultrasound group (Alginate + FITC-VAN emulsion) (*p* < 0.01, Figure 1d,f). Similarly, in fibrin hydrogels, Fibrin + FITC-VAN emulsion + US reached 25.6% ± 1.7% on Day 1 and 44% ± 2.8% by Day 7, compared to only 1% ± 0.3% and 1.6% ± 0.5% in the nontreated group (Fibrin + FITC-VAN emulsion) (*p* < 0.001, Figure 1e,f).

Fluorometric analysis showed similar release trends to standard VAN, with lower absolute values. FITC-VAN showed reduced cumulative release in both alginate and fibrin hydrogels compared to spectrophotometric measurements, while preserving the relative release behavior between hydrogel types (alginate/fibrin release ratio: 0.83 vs. 0.77 for VAN).

The emulsions effectively retain VAN and prevent its burst release. This corresponds to a relative release factor of approximately 0.30 for alginate and 0.35 for fibrin in the regular formulation, and 0.08 and 0.02 in the fluorescent formulation. It should be noted that the total quantity of VAN employed in the fluorescent assays was markedly lower (5 μg in 0.5 mL hydrogel) when compared to the standard release tests (250 μg in 1 mL). This substantial difference in initial drug loading likely contributed to the diminished release percentages observed in the fluorescent assays, as a reduced drug concentration within a delivery system is generally associated with a decreased driving force for diffusion (concentration gradient), thus leading to slower release kinetics.

Ultrasound stimulation enhanced drug release from emulsion-loaded alginate hydrogels by 1.83-fold for VAN and 8.75-fold for FITC-VAN compared to nontreated controls. In fibrin hydrogels, fluorescence-based analysis revealed a 27.5-fold increase in cumulative release following ultrasound application, indicating a stronger ultrasound responsiveness of fibrin relative to alginate under these conditions.

Figure 2 shows the cumulative release profile of VAN from emulsions over a 7-day period in deionized (DI) water, with samples stored at 4°C. The black line and data points represent emulsions maintained under static conditions without insonation. Throughout Days 0, 1, 3, and 7, the cumulative release remains negligible, staying close to 0%, indicating excellent drug retention by the emulsions in the absence of an external trigger. Conversely, the red line illustrates the release observed immediately following ultrasound application on Day 7 (Day 7 + US). A pronounced burst release is observed, with cumulative VAN release reaching approximately 27% (*p* < 0.0001). When incorporated into hydrogels, emulsions showed greater cumulative release, likely due to swelling of the hydrogel that promotes droplet destabilization and the higher incubation temperature (37°C vs. 4°C), which accelerates drug diffusion. These results confirm that while the emulsions remain highly stable under passive refrigerated conditions, ultrasound serves as a potent external stimulus capable of inducing substantial and rapid drug release, supporting their utility for controlled, on-demand therapeutic delivery.

### 3.2 ∣ Antibacterial Activity Assays

The antibacterial activity of VAN released from the hydrogels was further assessed using the Kirby–Bauer disk diffusion method [36]. Figure 3a shows representative MSSA agar plates with ZOIs produced by a 0.25 mg/mL VAN control and alginate and fibrin hydrogels assessed at multiple time points before and after ultrasound treatment.

Figure 3b highlights a pronounced burst release from alginate and fibrin hydrogels without emulsions on Day 1, with inhibition zones of 12.4 and 9.7 mm, respectively (*p* < 0.01). In ultrasound-treated emulsion-loaded alginate hydrogels, the ZOI increased from 7.8 mm on Day 1 to 8.4 mm by Day 7 and was significantly larger than the untreated emulsion group at Day 7 (*p* = 0.03), indicating delayed but ultrasound-enhanced VAN release. For the nonultrasound-treated emulsion-loaded alginate hydrogels (Alginate + VAN emulsion), the ZOI starts at 6.6 mm on Day 1, increases slightly to 6.9 mm by Day 3, and then decreases thereafter, indicating minimal release of active VAN. The changes over time in Alginate + VAN emulsion were not statistically significant (*p* > 0.05). This suggests that the hydrogel housing provides some shielding of the emulsion, reducing the amount of spontaneous nonultrasound-triggered release. Notably, no measurable ZOIs were observed in fibrin hydrogels with emulsions, regardless of ultrasound treatment, indicating that the released VAN did not reach concentrations sufficient to produce detectable antibacterial activity. This is likely due to interactions between VAN and the fibrin matrix, which can hinder drug diffusion and reduce its bioavailability at the assay interface. Together, these results emphasize the influence of hydrogel composition, loading method, and ultrasound triggering on the antibacterial efficacy of VAN release.

The stability of the VAN solution was evaluated by storing a 0.1 mg/mL preparation, chosen for its optimal performance in the Kirby–Bauer test, at 4°C and comparing ZOI measurements on Days 1 and 7. As shown in Figure 4, there was no significant difference (*p* = 0.4) between the two time points, indicating that the VAN solution remained stable during refrigerated storage.

Figure 5 presents the converted VAN concentrations (mg/mL) derived from the ZOI diameters using a standard calibration curve. This calibration curve (Figure 5a) was derived from ZOIs generated by known VAN concentrations ranging from 20 to 1000 μg/mL (Figure 5b). The curve was constructed using five replicates per concentration. On Day 1, the alginate hydrogel without emulsions (Alginate + VAN) exhibited an incremental release of 64%, indicating a significant burst release (Figure 5c). In contrast, the fibrin hydrogel without emulsions (Fibrin + VAN) showed a much lower incremental release of 32% (Figure 5d). For both of these directly loaded groups, the incremental release concentrations decreased steadily over time following the initial burst (Alginate + VAN: Day 1 vs. Day 7, *p* < 0.01, Fibrin + VAN: Day 1 vs. Day 7, *p* < 0.001). In the emulsion-loaded alginate hydrogel with ultrasound treatment (Alginate + VAN emulsion + US), the converted VAN concentration on Day 1 was 6%, which gradually increased to 8% by Day 7, reflecting a sustained and ultrasound-enhanced release (statistically not significant, *p* = 0.56, Figure 5c). In the absence of ultrasound (Alginate + VAN emulsion), the release concentration on Day 1 was 2%, with minimal changes over the subsequent days. For the emulsion-loaded fibrin hydrogels (Fibrin + VAN emulsion and Fibrin + VAN emulsion + US), the converted VAN concentration remained negligible throughout the 7-day period (< 0.6%), indicating limited or no effective drug release under those conditions (Figure 5d). Figure 5e,f compares VAN concentration estimates derived from inhibition zone measurements with those obtained through spectrophotometric analysis. Both methods yielded similar release trends across all groups. The differences between emulsion-loaded alginate and directly VAN-loaded alginate were not statistically significant (*p* > 0.05). However, for ultrasound-treated emulsion-loaded alginate, the differences were significant (*p* < 0.05). For fibrin hydrogels, differences were significant across most groups (*p* < 0.05), likely due to limited antibacterial activity and interference from the fibrin matrix in spectrophotometric measurements, leading to elevated absorbance values.

### 3.3 ∣ Macroscopic Integrity of Hydrogels

Figure 6 presents the appearance of various alginate and fibrin hydrogel groups after 7 days in medium. As shown in the figure, the alginate-based hydrogels exhibited more pronounced degradation compared to the fibrin-based groups, as indicated by the reduction in size of the sample. Notably, Alginate + VAN emulsion + US, which corresponds to ultrasound-treated alginate, showed the most severe degradation. In this group, only one sample retained its structure and could be retrieved from the medium, while the others had completely disintegrated. Fibrin hydrogels, regardless of their loading method or insonation, maintained their macroscopic structural integrity after 7 days in DPBS. These results further demonstrate the tunability of this platform for various drug delivery applications, where a fast-disintegrating hydrogel may be preferable to reduce surfaces that may become a nidus for biofilm formation, while a slower-degrading hydrogel may be preferable for longer incubation and sustained release. Quantitative analysis of the mean hydrogel diameter (Figure 6c) showed a significant decrease after 7 days, with alginate exhibiting a greater reduction (*p* < 0.001) than fibrin (*p* < 0.05).

## 4 ∣ Discussion

Spinal fusion surgery requires hydrogel-based delivery systems capable of providing sustained VAN release during the early postoperative period, particularly while surgical drains are in place, to ensure sufficient concentration for continuous prophylaxis. Additionally, these systems should be able to be triggered for the release of a complementary antibiotic upon drain removal, using noninvasive external stimuli such as ultrasound. In this study, alginate and fibrin hydrogels with directly loaded VAN exhibited a bolus release profile, which is advantageous during the initial days postsurgery when high local antibiotic concentrations are critical to prevent bacterial adhesion, biofilm formation, and early infections. However, the drug concentration in these formulations declined rapidly after the first few days, indicating a need for supplemental systems that can maintain therapeutic levels over extended periods and allow on-demand release.

Emulsion-loaded hydrogels addressed this limitation by demonstrating a more sustained release profile. Importantly, drug release from these systems could be enhanced through ultrasound stimulation, allowing temporal control over antibiotic delivery. This dual-release approach: initial burst from directly loaded hydrogels followed by sustained, triggerable release from emulsion systems, aligns well with clinical demands for infection control in spinal fusion procedures. Overall, the findings of this in vitro study support the potential of integrating ultrasound-responsive hydrogel systems into surgical site infection prophylaxis strategies.

Alginate and fibrin were selected as hydrogel matrices for this dual drug delivery system due to their well-established biocompatibility, biodegradability, and responsiveness to ultrasound-mediated mechanical forces, features that make them particularly suitable for postoperative therapeutic applications [24, 25, 33, 34]. Initial experiments incorporating free VAN directly into both hydrogels revealed a pronounced burst release followed by near-complete drug release (cf. Figure 1a-c), with fibrin releasing significantly more than alginate (*p* = 0.02), likely due to its higher swelling ratio. Additionally, spectrophotometric analysis, particularly in fibrin-based samples, was compromised by hydrogel degradation products, which interfered with absorbance measurements and led to apparent overestimations of drug release. To overcome these limitations, FITC–VAN was used, enabling fluorescence-based quantification with greater specificity and reduced susceptibility to background interference (cf., Figure 1d-f).

The emulsions used in this study effectively retain their VAN payload and prevent its spontaneous burst release, especially when loaded within the hydrogel. This behavior can be attributed to the structural integrity of the emulsions, which likely act as physical barriers limiting immediate drug diffusion. Several studies have shown that encapsulating hydrophilic drugs like VAN in oil-in-water emulsions or lipid-based carriers can reduce initial burst release and enable more controlled, sustained release profiles [25, 26, 37-39]. However, we did observe substantial differences in initial drug loading and corresponding release kinetics between the spectrophotometric and fluorometric assays (Figure 1). These differences in loading likely contributed to the diminished release percentages observed in the fluorescent assays, as a reduced drug concentration within a delivery system is generally associated with a decreased driving force for diffusion, thus leading to slower release kinetics. This phenomenon is consistent with principles of diffusion, where the rate is directly proportional to the concentration gradient across the release medium [40]. Nonetheless, in both testing conditions, the reduction in drug release compared to directly loaded hydrogels was substantial and statistically significant (*p* < 0.05). These findings underscore the effectiveness of the emulsion in retaining the VAN payload within the hydrogel matrix and minimizing the initial burst release. However, maintaining drug levels within the therapeutic window and enabling controlled, on-demand release, particularly after surgical drain removal, are critical.

The incorporation of ultrasound as a release trigger further strengthens the potential clinical utility of this system by enabling precise temporal control over drug release. Results involving FITC-VAN demonstrated a significant increase in drug release from emulsion-loaded hydrogels following insonation (*p* < 0.0001; Figure 1d-f). This enhancement is primarily attributed to ADV, a process initiated when the applied acoustic pressure surpasses a critical threshold, which for perfluorohexane is 2.2 ± 0.2 MPa at 2.5 MHz [41]. During the negative pressure phase of the ultrasound wave, perfluorohexane emulsions undergo a liquid-to-gas phase transition, creating mechanical disruption within the hydrogel matrix and facilitating the rupture of the emulsion shell [25, 26]. This disruption allows for localized and controlled release of the encapsulated drug. Such ultrasound-responsive behavior enables externally tunable release kinetics, offering the potential for noninvasive, on-demand antibiotic delivery tailored to postoperative needs.

The observed differences in VAN release between spectrophotometric and fluorescent assays warrant consideration. The fluorescent assays employed a 25-fold lower initial VAN concentration (5 μg in 0.5 mL hydrogel) than the spectrophotometric assays (250 μg in 1 mL), due to the reduced solubility of FITC-VAN and the limited dynamic range of fluorescence measurements, where quenching can occur at higher concentrations. According to Fick′s First Law of Diffusion, this lower concentration gradient likely contributed to the reduced release percentages observed in the fluorescent assays. Although both methods aimed to maintain sink conditions, deviations from ideality at low drug loading may have further influenced release kinetics [42, 43]. In addition, spectrophotometric measurements are susceptible to interference from hydrogel degradation products, whereas fluorescence-based detection, while more specific, can be affected by inner filter effects and quenching within the matrix [44-46]. Future studies could normalize release profiles to initial drug loading and incorporate complementary techniques such as HPLC to further validate these findings.

To further support our findings and evaluate the antibacterial efficacy of various hydrogel formulations, the Kirby–Bauer disk diffusion assay was utilized. It is important to note that the accuracy of the Kirby–Bauer test can decrease at higher antibiotic concentrations due to saturation and limited diffusion [47]. While spectrophotometric and fluorescence measurements indicated substantial VAN release from fibrin hydrogels, particularly with ultrasound stimulation, the Kirby–Bauer assays revealed limited or no corresponding antibacterial effect. This apparent paradox may be attributed to several factors, including the potential for VAN to interact with components of the fibrin matrix, thereby reducing its bioavailability and diffusion. Fibrin, a complex proteinaceous network, is known to interact with various molecules, and such interactions can hinder the diffusion of released drugs through the hydrogel and into the surrounding medium, ultimately limiting their effectiveness in inhibiting bacterial growth. These interactions highlight the importance of considering drug-matrix interactions in the design of hydrogel-based delivery systems, as they can significantly influence the therapeutic efficacy of released drugs [48-50]. Rather than delivering a rapid, high-dose burst, the emulsion system provided prolonged release within a therapeutic window, allowing for continuous antimicrobial activity. This release profile is particularly desirable in clinical contexts where persister or biofilm-associated bacteria are present, as these subpopulations are more tolerant to antibiotics and often evade short-term high-dose regimens. Sustained exposure at sub-MIC (minimum inhibitory concentration) or near-MIC levels over several days has been shown to enhance bacterial eradication, especially in difficult-to-treat infections [22, 51]. MIC refers to the lowest concentration of an antibiotic required to inhibit visible bacterial growth. For VAN against *Staphylococcus aureus*, MIC values typically range from ≤ 2 μg/mL for susceptible strains to > 16 μg/mL in resistant strains [52-54]. Maintaining drug levels around this threshold is crucial for suppressing bacterial survival and preventing regrowth. Thus, the ultrasound-responsive alginate system offers a promising strategy for combating infections in high-risk postsurgical sites.

ZOI diameters were converted to VAN concentrations using a calibration curve, yielding release profiles (Figure 5) that closely matched spectrophotometric data (Figure 1). However, with the exception of the Alginate + VAN group, the concentrations estimated from ZOI data are generally lower than those measured by spectrophotometry. As discussed above, optical interference may affect spectrophotometric readings. In contrast, Alginate + VAN shows a higher converted concentration than the spectrophotometric value. This anomaly may result from the reduced precision of the Kirby–Bauer method at higher VAN concentrations, where the inhibition zone plateaus due to saturation effects. Given that the calibration curve follows a logarithmic relationship, small increases in zone diameter correspond to disproportionately large increases in calculated concentration at higher values, making the method less reliable in that range. This limitation underscores the need for careful interpretation of ZOI-derived concentrations, especially at elevated drug levels.

After 7 days in media (Figure 6), fibrin hydrogels maintained structural integrity regardless of VAN loading or insonation, whereas alginate, especially ultrasound-treated emulsion-loaded samples, showed pronounced degradation, consistent with cavitation-induced stresses. This differential stability underscores a critical consideration for clinical application, while enhanced structural stability, as demonstrated by fibrin, may be beneficial for applications requiring prolonged material presence; there are scenarios where rapid degradation is actually advantageous. For instance, a fast-dissolving drug delivery vehicle can prevent complications associated with residual foreign materials in confined anatomical spaces such as the spine. Thus, the observed differences in degradation profiles underscore a key strength of our hydrogel system: tunability. By selecting or modifying hydrogel composition, such as adjusting crosslinking density or incorporating stabilizing components, the platform can be tailored to support either extended residence time or rapid clearance, depending on the clinical context and therapeutic goals.

A limitation of this work is that although fibrin hydrogels achieved favorable release profiles, their weak antibacterial efficacy may reduce clinical utility. Similarly, while alginate hydrogels demonstrated superior antibacterial performance, their structural integrity was compromised when combined with emulsion droplets, raising concerns for extended treatments. In addition, vancomycin was used only as a model drug; evaluating the platform with other clinically relevant antibiotics, particularly those effective against biofilm-forming or resistant pathogens, could broaden its translational relevance. Future studies should explore strategies such as surface coatings, nanoparticle incorporation, or enhanced crosslinking to improve the stability and efficacy of both systems for long-term, responsive antibiotic delivery.

Overall, the key contribution of this work is the demonstration of a tunable, dual-phase hydrogel platform that combines early bolus dosing with sustained, ultrasound-triggered antibiotic release tailored to the risk of postsurgical infection.

## 5 ∣ Conclusion

Spinal fusion procedures face a persistent risk of bacterial infection due to biofilm formation after drain removal and depletion of local antibiotics. This study introduces a composite hydrogel-based delivery platform capable of immediate and controlled antibiotic release. Directly loaded hydrogels ensure high initial VAN levels critical for early-stage prophylaxis, while emulsion-embedded systems allow extended retention and ultrasound-triggered on-demand release. Emulsion-loaded alginate hydrogels, in particular, demonstrate strong potential for therapeutic use, achieving effective drug release and antibacterial activity upon stimulation.

Insonation significantly enhanced drug release in alginate and fibrin matrices, though fibrin-based emulsions required further optimization to translate release into detectable antibacterial activity. The dual-mode system aligns well with clinical needs: delivering an early therapeutic burst followed by spatially controllable release during the vulnerable postdrain removal period. These findings support the continued development of ultrasound-responsive hydrogel systems for preventing implant-associated infections.

## Supplementary Material

Suppl.

Additional supporting information can be found online in the Supporting Information section. **Data S1:** jbma70046-sup-0001-Supplementary_data.docx.

## Figures and Tables

**FIGURE 1 ∣:**
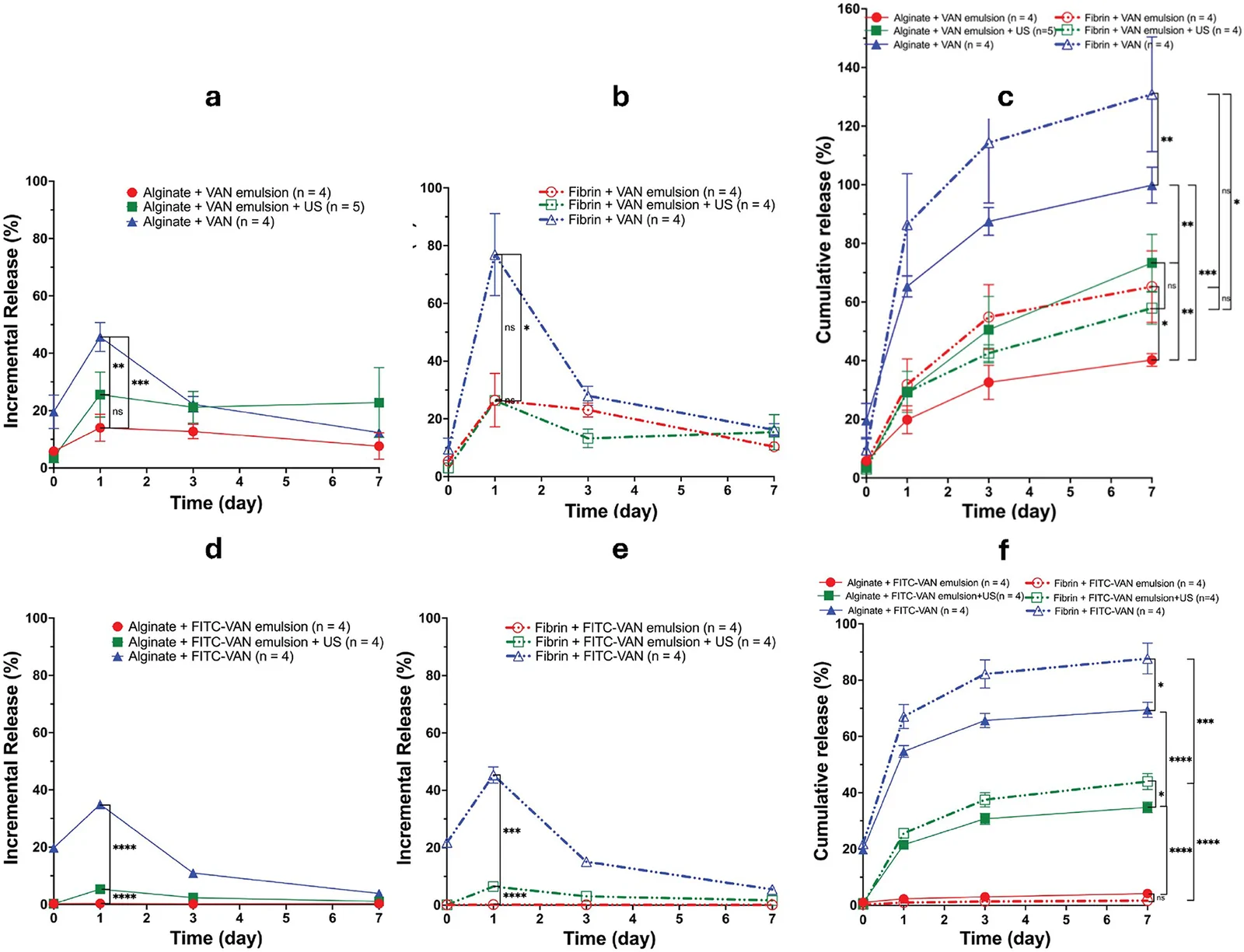
Incremental (a, b, d, and e) and cumulative (c and f) release profiles of VAN (a–c) and FITC-VAN (d–f) from alginate (a and d) and fibrin (b and e) hydrogels. This figure demonstrates the comparative release behavior of VAN and FITC–VAN from different hydrogel formulations. Significant differences are indicated as follows: *p < 0.05; **p < 0.01; ***p < 0.001; ****p < 0.0001; ns: not significant. Data show mean ± SD (*n* ≥ 4). Individual data points are shown in Figure S1.

**FIGURE 2 ∣:**
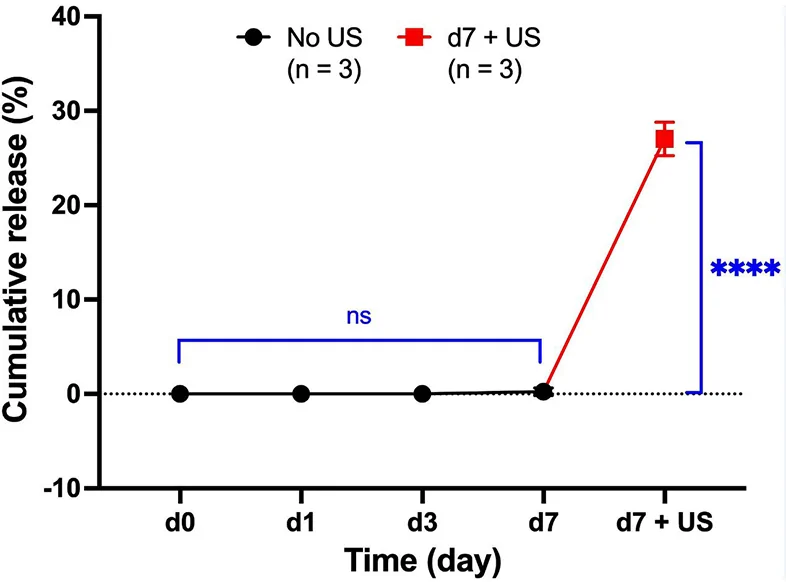
Ultrasound triggers rapid VAN release from stable emulsions in DI water over 7 days, demonstrating their potential for on-demand drug delivery. Significant differences are indicated as follows: ****p < 0.0001; ns: not significant. Data are presented as mean ± SD (*n* = 3). Individual data points are shown in Figure S2.

**FIGURE 3 ∣:**
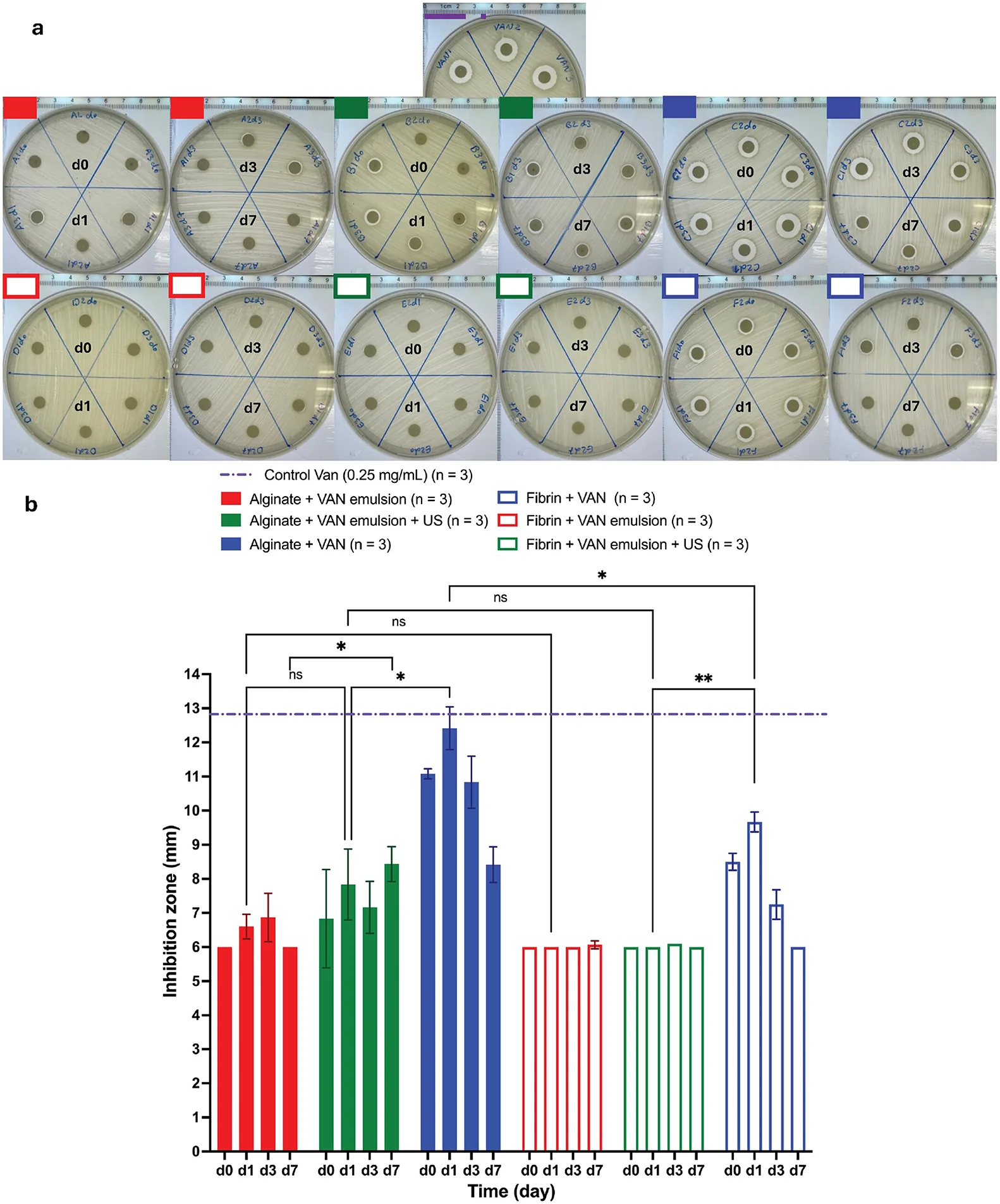
Sustained inhibition of MSSA growth by VAN released from alginate and fibrin hydrogels over 7 days. (a) Representative agar diffusion assays and (b) corresponding inhibition zone diameters demonstrate prolonged antibacterial activity compared with the VAN solution control. Significant differences are indicated as follows: *p < 0.05; **p < 0.01; ***p < 0.001; ****p < 0.0001. Nonsignificant differences are denoted as ns. Data are presented as mean ± SD (*n* = 3). Individual data points are shown in Figure S3.

**FIGURE 4 ∣:**
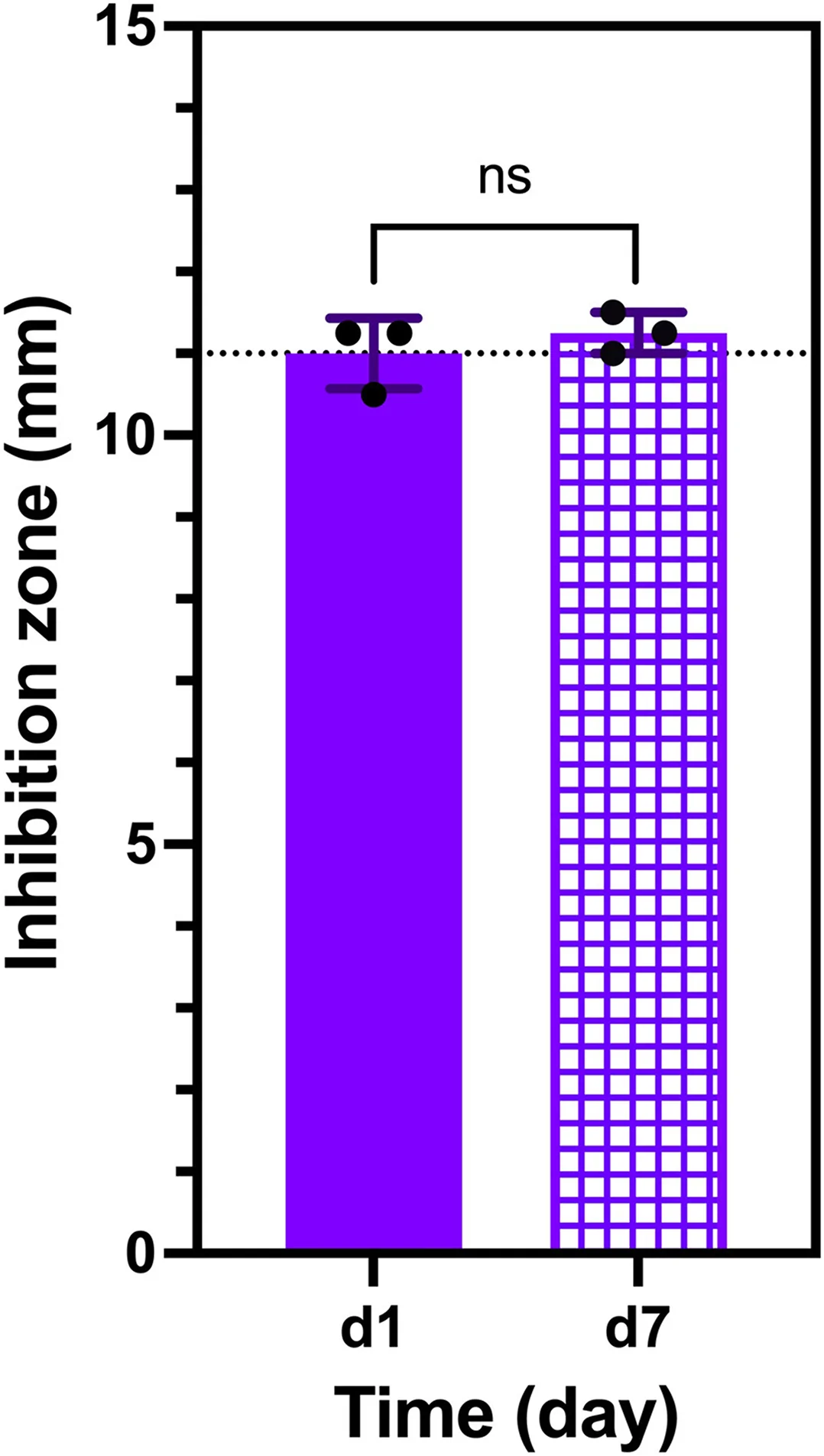
The ZOI of a 0.1 mg/mL VAN solution after 7 days of refrigerated storage shows no significant change, confirming the drug′s stability. ns = not significant. Data are presented as mean ± SD (*n* = 3). Individual data points are shown in Figure S4.

**FIGURE 5 ∣:**
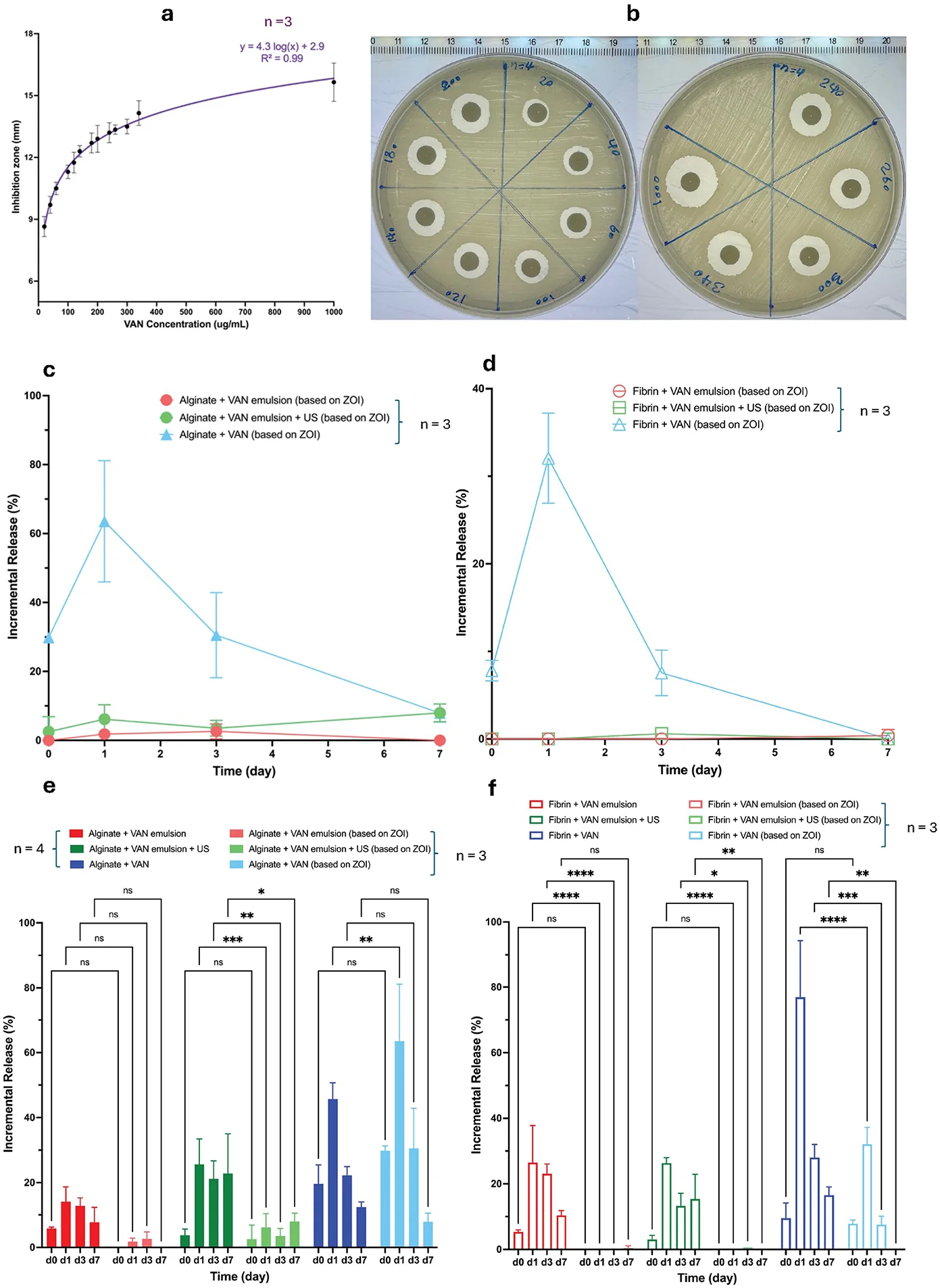
VAN concentrations estimated from inhibition zone diameters using a standard calibration curve (a and b) for alginate (c) and fibrin (d) hydrogels, and their comparison with spectrophotometric measurements at corresponding time points (e and f). The data illustrate formulation- and ultrasound-dependent differences in VAN release profiles. Significant differences are indicated as follows: *p < 0.05; **p < 0.01; ***p < 0.001; ****p < 0.0001. Nonsignificant differences are denoted as ns. Data are presented as mean ± SD (*n* ≥ 3). Individual data points are shown in Figure S5.

**FIGURE 6 ∣:**
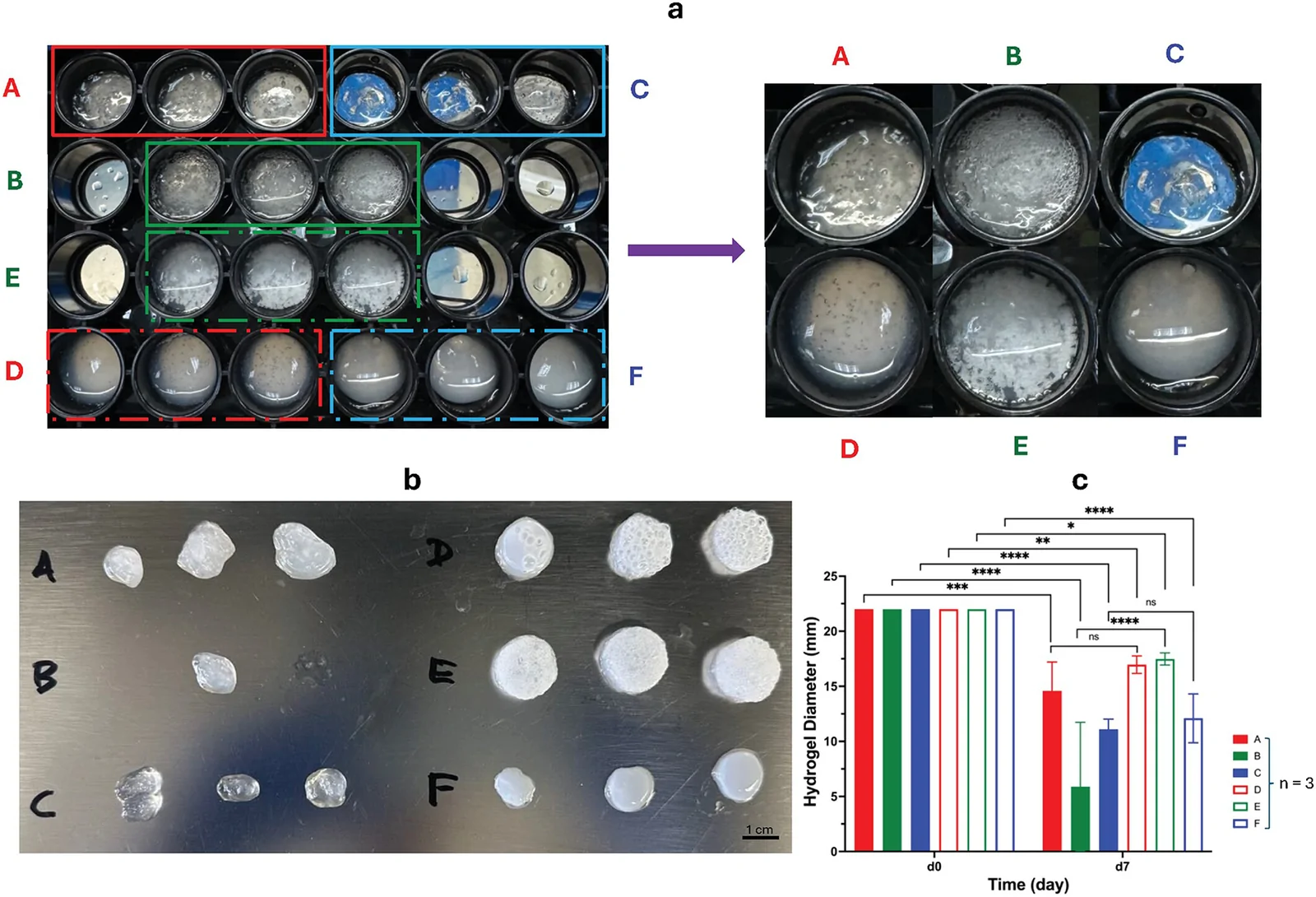
Macroscopic stability of alginate and fibrin hydrogels with and without emulsions and ultrasound treatment. Representative images at Day 0 and after 7 days in DPBS (a and b) and corresponding changes in mean hydrogel diameter (c) show that all formulations remain intact, with fibrin hydrogels exhibiting greater dimensional stability. Significant differences are indicated as follows: *p < 0.05; **p < 0.01; ***p < 0.001; ****p < 0.0001. Nonsignificant differences are denoted as ns. Data are presented as mean ± SD (*n* = 3). Individual data points are shown in Figure S6.

**TABLE 1 ∣:** Experimental groups—alginate and fibrin hydrogels.

Group	VAN loading method	Ultrasound (US) treatment
Alginate + VAN	Direct loading (free VAN)	No
Alginate + VAN emulsion + US	Emulsion-loaded (encapsulated VAN)	Yes (applied Day 0)
Alginate + VAN emulsion	Emulsion-loaded (encapsulated VAN)	No
Fibrin + VAN	Direct loading (free VAN)	No
Fibrin + VAN emulsion + US	Emulsion-loaded (encapsulated VAN)	Yes (applied Day 0)
Fibrin + VAN emulsion	Emulsion-loaded (encapsulated VAN)	No

## Data Availability

The data that support the findings of this study are available on request from the corresponding author. The data are not publicly available due to privacy or ethical restrictions.
