# Supplementary material for: Controlled Antibiotic Release From Emulsion-Loaded Alginate and Fibrin Hydrogels Using Ultrasound
Source: J Biomed Mater Res A. Author manuscript; Available in PMC 2026 Oct 5. (PMC13636844; doi:10.1002/jbma.70046)
Supplement: Supplementary file 1 [file NIHMS2212886-supplement-Suppl_.docx]

**a**

**b**

**c**

**d**

**e**

**f**

Fig S1. Incremental (a, b, d, e) and cumulative (c, f) release profiles of VAN (a–c) and FITC-VAN (d–f) from alginate (a, d) and fibrin (b, e) hydrogels based on individual data points.

Fig S2. Ultrasound triggers rapid VAN release from stable emulsions in DI water over 7 days, demonstrating their potential for on-demand drug delivery. Data are presented as individual data points.

Fig S3. Sustained inhibition of MSSA growth by VAN released from alginate and fibrin hydrogels over 7 days. Corresponding inhibition zone diameters demonstrate prolonged antibacterial activity compared with the VAN solution control. Data are presented as individual data points.

Fig S4. The ZOI of a 0.1 mg/mL VAN solution after 7 days of refrigerated storage shows no significant change, confirming the drug's stability. Data are presented as individual data points.

**c**

**d**

**e**

**f**

**a**

Fig S5. VAN concentrations estimated from inhibition zone diameters using a standard calibration curve (a) for alginate (c) and fibrin (d) hydrogels, and their comparison with spectrophotometric measurements at corresponding time points (e,f). The data illustrate formulation- and ultrasound-dependent differences in VAN release profiles. Data are presented as individual data points.

Fig S6. Quantitative Stability of alginate and fibrin hydrogels with and without emulsions and ultrasound treatment. Data are presented as individual data points.

Table S 1. Summary of VAN and FITC-VAN release from hydrogel formulations.

| **Group** | **n** | **Loading Method** | **Ultrasound (US)** | **Day 1 Release (%)** | **Day 7 Cumulative Release (%)** | **Key Observation / Comparison** |
| --- | --- | --- | --- | --- | --- | --- |
| Alginate + VAN emulsion | 4 | Emulsion | No | 20 ± 5 % | 40 ± 2 % | Reduced burst, more sustained release |
| Alginate + VAN emulsion + US | 5 | Emulsion | Yes (day 0) | 29 ± 7 % | 73 ± 10 % | Ultrasound nearly doubles release (*p* < 0.01) |
| Alginate + VAN | 4 | Free VAN | No | 65 ± 4 % | 100 ± 6 % | Strong burst release, complete by day 7 |
| Fibrin + VAN emulsion | 4 | Emulsion | No | 32 ± 11 % | 65 ± 15 % | Moderate sustained release |
| Fibrin + VAN emulsion + US | 4 | Emulsion | Yes (day 0) | 29 ± 1 % | 58 ± 7 % | No statically significant effect of ultrasound (*p* > 0.05) |
| Fibrin + VAN | 4 | Free VAN | No | 86 ± 21 % | 130 ± 24 % | Apparent >100% release due to degradation/interference |
| Alginate + FITC-VAN emulsion | 4 | Emulsion | No | 2.3 ± 0.3 % | 4.2 ± 1.2 % | Minimal release, strong retention |
| Alginate + FITC-VAN emulsion + US | 4 | Emulsion | Yes (day 0) | 22 ± 1 % | 35 ± 2 % | ultrasound enhanced release (~8× higher vs. no US, p < 0.01) |
| Alginate + FITC-VAN | 4 | Free FITC-VAN | No | 55 ± 2 % | 69 ± 2.7 % | Burst release, moderate cumulative release |
| Fibrin + FITC-VAN emulsion | 4 | Emulsion | No | 1 ± 0.3 % | 1.6 ± 0.5 % | Negligible release without US |
| Fibrin + FITC-VAN emulsion + US | 4 | Emulsion | Yes (day 0) | 25.6 ± 1.7 % | 44 ± 2.8 % | Strong ultrasound effect (~25–30× higher vs. no US, p < 0.001) |
| Fibrin + FITC-VAN | 4 | Free FITC-VAN | No | 67 ± 4.3 % | 88 ± 5.5 % | Higher release vs. alginate (p = 0.02) |
